# Supplementary material for: Using laser micro-dissection and qRT-PCR to analyze cell type-specific gene expression in Norway spruce phloem
Source: PeerJ. 2014 Apr 29;2:e362. doi: 10.7717/peerj.362 (PMC4017884; doi:10.7717/peerj.362)
Supplement: Supplemental Information 1 — Gene expression was determined in sections taken 5 and 10 mm above the inoculation site in ramet A and B of clone 471. Control samples are from unharmed tissues of ramet A, C and D of clone 471. Data are presented as relative transcript abundance normalized to actin expression. Dash (—), indicates that the sample was not subjected to target gene profiling due to low RNA yield (cycle threshold value for actin above 35). [file peerj-02-362-s001.pdf]

Gene	Tissue and cells	Infected				Control			
		Ramet A 5mm d3 2003	Ramet B 5mm d3 2003	Ramet A 10mm d3 2003	Ramet B 10mm d3 2003	Ramet A site 1 d35 2003	Ramet A site 2 d35 2003	Ramet C 2010	Ramet D 2010
CHI4	Primary phloem	3.03	0.86	-	1.38	0.00	0.01	-	0.00
	Sec. phloem conducting	19.52	6.78	1.32	1.74	0.00	0.04	0.00	-
	Sec. phloem non-conducting	49.84	2.45	1.76	0.60	0.00	0.00	0.00	0.00
	Cambium	5.21	0.41	1.03	-	0.20	0.02	0.00	0.00
	Ray cells	51.66	4.36	3.87	0.49	0.00	0.00	0.00	0.00
	PP cells	29.79	2.35	3.64	0.30	0.00	3.71	0.00	0.00
PAL	Primary phloem	3.72	2.23	-	1.68	0.02	0.05	-	0.03
	Sec. phloem conducting	2.44	4.34	1.89	0.81	0.14	0.14	0.01	-
	Sec. phloem non-conducting	7.42	3.20	2.07	0.87	0.03	0.02	0.001	0.02
	Cambium	6.09	2.62	1.09	-	0.08	0.05	0.08	0.06
	Ray cells	8.21	4.27	4.85	1.89	0.00	0.00	0.02	0.00
	PP cells	3.65	1.60	6.42	2.33	0.00	0.38	0.00	0.00
SPI1	Primary phloem	0.81	0.00	-	0.00	0.00	0.00	-	0.15
	Sec. phloem conducting	0.37	0.00	2.54	0.80	1.32	0.42	0.14	-
	Sec. phloem non-conducting	0.41	0.53	2.18	0.11	2.48	0.31	0.00	0.36
	Cambium	0.36	4.13	4.31	-	0.00	0.00	2.19	0.65
	Ray cells	3.96	2.29	5.74	5.35	0.00	0.00	1.80	2.15
	PP cells	0.46	0.34	0.47	1.34	0.00	0.00	0.00	0.00
PX3	Primary phloem	0.07	0.10	-	0.05	0.00	0.01	-	0.001
	Sec. phloem conducting	0.24	1.22	0.12	0.83	0.00	0.00	0.02	-
	Sec. phloem non-conducting	0.33	0.53	0.39	0.16	0.00	0.00	0.00	0.00
	Cambium	0.37	13.73	0.43	-	0.94	0.00	0.03	0.00
	Ray cells	1.90	11.07	0.38	1.03	0.00	0.00	0.00	0.00
	PP cells	0.06	0.18	0.00	0.11	0.00	0.00	0.00	0.00
TIF	Primary phloem	1.59	0.90	-	0.71	0.76	0.48	-	1.82
	Sec. phloem conducting	0.99	0.81	1.17	0.59	3.11	0.70	1.43	-
	Sec. phloem non-conducting	2.18	0.68	1.32	0.62	0.94	0.60	0.97	1.69
	Cambium	1.28	2.59	1.10	-	2.84	1.13	1.65	1.67
	Ray cells	3.28	2.65	1.75	1.82	0.69	1.03	1.15	3.22
	PP cells	2.20	0.67	0.83	1.55	0.00	0.00	1.05	3.18

Ramet = trees of Norway spruce clone number 471; mm = distance from inoculation site; d = day
