# Supplementary material for: Using laser micro-dissection and qRT-PCR to analyze cell type-specific gene expression in Norway spruce phloem
Source: PeerJ. 2014 Apr 29;2:e362. doi: 10.7717/peerj.362 (PMC4017884; doi:10.7717/peerj.362)
Supplement: Supplemental Information 2 — Gene expression was determined in sections taken 5 and 10 mm above the inoculation site in ramet A and B of clone 471. Data are presented as relative transcript abundance normalized to actin expression. Dash (—), indicates that the sample was not subjected to target gene profiling due to low RNA yield (cycle threshold value for actin above 35). [file peerj-02-362-s002.docx]

|  |  |  | | | |  |  | |  |  |
| --- | --- | --- | --- | --- | --- | --- | --- | --- | --- | --- |
|  |  | **Infected** | | | |  | **Control** | |  |  |
|  |  |  |  |  |  |  |  |  | |  |
| **Gene** | **Tissue and cells** | **Ramet A 5mm d3** | **Ramet A 10mm d3** | **Ramet B 5mm d3** | **Ramet B 10mm d3** |  | **Ramet A site1 d35** | **Ramet A site2 d35** | |  |
| CHI4 | Primary phloem | 3.03 | **-** | 0.86 | 1.38 |  | 0.00 | 0.01 | |  |
|  | Sec. phloem conducting | 19.52 | 1.32 | 6.78 | 1.74 |  | 0.00 | 0.04 | |  |
|  | Sec. phloem non-conducting | 49.84 | 1.76 | 2.45 | 0.60 |  | 0.00 | 0.00 | |  |
|  | Cambium | 5.21 | 1.03 | 0.41 | **-** |  | 0.20 | 0.02 | |  |
|  | Ray cells | 51.66 | 3.87 | 4.36 | 0.49 |  | 0.00 | 0.00 | |  |
|  | PP cells | 29.79 | 3.64 | 2.35 | 0.30 |  | 0.00 | 3.71 | |  |
| PAL | Primary phloem | 3.72 | **-** | 2.23 | 1.68 |  | 0.02 | 0.05 | |  |
|  | Sec. phloem conducting | 2.44 | 1.89 | 4.34 | 0.81 |  | 0.14 | 0.14 | |  |
|  | Sec. phloem non-conducting | 7.42 | 2.07 | 3.20 | 0.87 |  | 0.03 | 0.02 | |  |
|  | Cambium | 6.09 | 1.09 | 2.62 | **-** |  | 0.08 | 0.05 | |  |
|  | Ray cells | 8.21 | 4.85 | 4.27 | 1.89 |  | 0.00 | 0.00 | |  |
|  | PP cells | 3.65 | 6.42 | 1.60 | 2.33 |  | 0.00 | 0.38 | |  |
| SPI1 | Primary phloem | 0.81 | **-** | 0.00 | 0.00 |  | 0.00 | 0.00 | |  |
|  | Sec. phloem conducting | 0.37 | 2.54 | 0.00 | 0.80 |  | 1.32 | 0.42 | |  |
|  | Sec. phloem non-conducting | 0.41 | 2.18 | 0.53 | 0.11 |  | 2.48 | 0.31 | |  |
|  | Cambium | 0.36 | 4.31 | 4.13 | **-** |  | 0.00 | 0.00 | |  |
|  | Ray cells | 3.96 | 5.74 | 2.29 | 5.35 |  | 0.00 | 0.00 | |  |
|  | PP cells | 0.46 | 0.47 | 0.34 | 1.34 |  | 0.00 | 0.00 | |  |
| PX3 | Primary phloem | 0.07 | **-** | 0.10 | 0.05 |  | 0.00 | 0.01 | |  |
|  | Sec. phloem conducting | 0.24 | 0.12 | 1.22 | 0.83 |  | 0.00 | 0.00 | |  |
|  | Sec. phloem non-conducting | 0.33 | 0.39 | 0.53 | 0.16 |  | 0.00 | 0.00 | |  |
|  | Cambium | 0.37 | 0.43 | 13.73 | **-** |  | 0.94 | 0.00 | |  |
|  | Ray cells | 1.90 | 0.38 | 11.07 | 1.03 |  | 0.00 | 0.00 | |  |
|  | PP cells | 0.06 | 0.00 | 0.18 | 0.11 |  | 0.00 | 0.00 | |  |
| TIF | Primary phloem | 1.59 | **-** | 0.90 | 0.71 |  | 0.76 | 0.48 | |  |
|  | Sec. phloem conducting | 0.99 | 1.17 | 0.81 | 0.59 |  | 3.11 | 0.70 | |  |
|  | Sec. phloem non-conducting | 2.18 | 1.32 | 0.68 | 0.62 |  | 0.94 | 0.60 | |  |
|  | Cambium | 1.28 | 1.10 | 2.59 | **-** |  | 2.84 | 1.13 | |  |
|  | Ray cells | 3.28 | 1.75 | 2.65 | 1.82 |  | 0.69 | 1.03 | |  |
|  | PP cells | 2.20 | 0.83 | 0.67 | 1.55 |  | 0.00 | 0.00 | |  |
| Ramet = trees of Norway spruce clone number 471; d = day; mm = distance from inoculation site | | | | | | | | | | |

**Table 2 Expression profiles of five genes in different tissue regions and cell types of Norway spruce phloem, after inoculation with *Ceratocystis polonica* and in control.** Gene expression was determined in sections taken 5 and 10 mm above the inoculation site in ramet A and B of clone 471. Data are presented as relative transcript abundance normalized to actin expression. Dash (-), indicates that the sample was not subjected to target gene profiling due to low RNA yield (cycle threshold value for actin above 35).
